# Enhancing Spore Inactivation: Low-Intensity Pulsed Electric Field Combined with Ohmic Heating and Germinant Pretreatment

**DOI:** 10.3390/foods15020230

**Published:** 2026-01-08

**Authors:** Fei-Yue Xu, Hua-Xi Huang, Qing-Hui Wen, Lang-Hong Wang, Yan-Yan Huang, Man-Sheng Wang

**Affiliations:** 1 Guangdong Provincial Key Laboratory of Intelligent Food Manufacturing, School of Food Science and Engineering, Foshan University, Foshan 528225, China; feiyuexu@fosu.edu.cn (F.-Y.X.); 14767786114@163.com (H.-X.H.); huang_yanyan@fosu.edu.cn (Y.-Y.H.); 2 School of Health, Jiangxi Normal University, Nanchang 330022, China; qinghuiwen@jxnu.edu.cn; 3 Institute of Bast Fiber Crops, Chinese Academy of Agricultural Sciences, Changsha 410205, China

**Keywords:** *Alicyclobacillus acidoterrestris*, pulsed electric field, germinants, reactive oxygen species

## Abstract

Bacterial spores, as one of the most resistant microbial forms, are difficult to completely eliminate through conventional heat treatments such as pasteurization, allowing them to persist in food and pose a significant threat to microbial safety. This study employed a “germination–inactivation” strategy to inactivate *Alicyclobacillus acidoterrestris* (AAT) spores using a germinant under low-intensity pulsed electric fields (PEFs). Analysis of germination curves identified 40 mM L-valine as the most effective germinant. Results showed that after 4-h incubation with 40 mM L-valine followed by 210 s of 0.18 kV/cm PEF treatment, the synergistic effect of electric field and ohmic heating (OH) reduced AAT spore counts by 1.73 log units. In contrast, the control group treated with the same PEF parameters without a germinant showed only a 0.54 log unit reduction. These findings indicate that germination agents significantly reduce spore resistance. Subsequent experiments confirmed that L-valine-treated AAT spores underwent pronounced structural disruption under the combined effects of the electric field and OH, leading to leakage of intracellular components such as nucleic acids and proteins. This phenomenon was verified via scanning electron microscopy (SEM) and laser confocal microscopy. Additionally, both ROS levels and ATPase activity in spores were substantially reduced, further indicating that the combined electric field and OH synergistically disrupted the spore’s external structure and internal macromolecules, leading to spore death. Thus, low-intensity PEF assisted by spore germination agents offers an energy-efficient and effective inactivation method, opening new avenues for spore inactivation research.

## 1. Introduction

*Alicyclobacillus acidoterrestris* (AAT) is a thermophilic, acidophilic, spore-forming bacterium commonly found in soil and frequently encountered in juice beverage production lines [1,2]. Its vegetative cells grow optimally at temperatures of 40–45 °C and at pH values of 3.5–4.0, with the capacity to survive at temperatures as high as 65 °C. Under unfavorable environmental conditions, AAT forms highly resistant spores capable of withstanding extreme conditions such as humidity, heat, and desiccation, allowing them to bypass pasteurization and contaminate juice products. When the living conditions become favorable, these spores germinate into vegetative cells that resume metabolism and reproduction [3]. During this process, the formation of guaiacol (2-methoxyphenol), a metabolic byproduct, imparts a pungent odor and induces sedimentation in the juice, thereby reducing its overall quality and market value [4].

Following germination, spores exhibit substantially diminished resistance to harsh environmental conditions, making them more amenable to inactivation by various sterilization methods. Germination factors, primarily small molecules, are generally classified as nutrient or non-nutrient. They play a crucial role in triggering spore germination and subsequently forming vegetative cells. Nutrient germination factors (e.g., amino acids, sugars) initiate the germination process by binding to specific germination receptors on the spore’s inner membrane, thereby driving the spore into its commitment phase. This phase includes the release of Ca-DPA (calcium dipicolinate), which activates cortical hydrolases, leading to cortex degradation and the gradual hydration of the spore core [5]. Concurrently, macromolecule synthesis and metabolic processes begin, culminating in the growth and proliferation of germinated spores. In contrast, non-nutrient germination factors facilitate later stages of germination by activating specific steps in the nutrient-driven pathway (for example, CaDPA can promote germination completion by activating the cortex hydrolase CwlJ) [6].

Research indicates that holding spores at 121 °C for over five minutes can effectively kill them [7]. However, such high temperatures can destroy heat-sensitive components (e.g., polyphenols, vitamins) in foods like fruit juices, leading to a significant decline in product quality and flavor [8,9]. In recent years, pulsed electric field (PEF) has stood out as a particularly promising approach for controlling microbial contamination through the principle of “electroporation”, in which an electric field is applied to biological cells or tissues, increasing membrane permeability. This process results in the formation of irreversible pores in the cell membrane and facilitates the release of intracellular substances, ultimately resulting in cell death [10,11].

Compared with vegetative cells, bacterial spores exhibit considerably higher resistance to environmental stressors, making their inactivation more challenging in the food industry [12]. Nonetheless, studies indicate that pulsed electric field (PEF) treatment can effectively reduce spore populations through a combination of thermal (Ohmic heating, OH, the direct conversion of electrical energy into thermal energy within a conductive material) and non-thermal (electroporation) mechanisms [13]. For example, Siemer et al. achieved a 4.5-log reduction in Bacillus subtilis PS832 spores at an electric field strength of 9 kV/cm [14]. Similarly, Choi et al. demonstrated that PEF treatment at a pulse intensity above 110 kV/cm and a pulse width of 100 ns reduced B. subtilis spores by up to 6.7 logs [15]. These findings confirm PEF as a promising technique for the inactivation of bacterial spores. Electroporation-induced cell damage depends largely on the electric field strength and the duration of exposure. Higher electric field strengths typically yield substantially greater spore inactivation rates [9,16]; however, the high cost of electricity has limited the widespread application of this technology in the food industry. Meanwhile, moderate-intensity pulsed electric field (PEF) treatment often proves inadequate for spore reduction. For example, Bacillus subtilis spores subjected to a moderate electric field (300 V/cm, below 30 °C) displayed only a 0.6-log reduction. Despite the synergistic interaction between the pulsed electric field and the accompanying ohmic heating effect, microbial inactivation efficiency was only moderately improved [7]. As spores enter the germination phase, their resistance to environmental stressors decreases, making them more vulnerable to inactivation. This observation forms the basis of the “germinate-to-eradicate” strategy, which induces germination prior to treatment and can enhance spore inactivation efficiency [17,18,19,20]. However, to date, few studies have evaluated the synergistic effects of germinants and PEF treatment on the spores of AAT.

Accordingly, this study aimed to examine the effects of various germinants and their concentrations on AAT spore germination, as well as to investigate the inactivating potential of combining these germinants with pulsed electric field (PEF) under uncontrolled temperature conditions (from 22 ± 0.61 °C to 78.7 ± 3.4 °C). Furthermore, the possible underlying mechanisms of PEF-induced spore inactivation were preliminarily explored.

## 2. Materials and Methods

### 2.1. Bacterial Strains and Instruments

The A. acidoterrestris ATCC 49025 strain was obtained from the Guangdong Microbial Strain Conservation Center (GDMCC) and preserved at −20 °C. Pulsed Electric Field Discharge Equipment (EX-1900) from Guangzhou Paihu Technology Co. (Guangzhou, China), UV–Visible Spectrophotometer (UV755B) from Shanghai Youke Instruments Co. (Shanghai, China), Scanning Electron Microscope (S-4800) from Hitachi, Japan Co., Ltd. (Tokyo, Japan), and Laser Confocal Microscopes (LSM800) from Carl Zeiss AG (Oberkochen, Germany). were used in this research.

### 2.2. Formation Process of AAT Spores and Purification

The *Alicyclobacillus acidoterrestris* medium (AAM), which is conducive to AAT growth, is prepared by dissolving 2 g glucose, 2 g yeast powder, 0.38 g anhydrous CaCl_2_, 1.2 g KH_2_PO_4_, 1 g MgSO_4_·7H_2_O, 0.38 g Mn_2_SO_4_·1H_2_O, and 0.4 g (NH_4_)_2_SO_4_, in ultrapure water to a final volume of 1 L and adjusting the pH to 4.0 [21].

The overnight activated AAT was transferred to the new AAM liquid medium and incubated in a shaker at 45 °C and 180 rpm for 3 days to produce AAT spores. The presence of spores was verified by microscopic examination. When the proportion of spores exhibiting typical strong refraction and full morphology reaches or exceeds 90% of the total cell count in the field of view, the desired maturity is considered achieved. At this point, spores were harvested and resuspended in sterile water. To purify the spores, the spore suspension was heated in a water bath at 80 °C for 20 min to inactivate the vegetative cells. The suspension was centrifuged at 4 °C and 6000 rpm for 8 min and then washed repeatedly with sterile physiological saline at least three times until microscopic examination revealed that most vegetative cell fragments were completely cleared, yielding a highly purified spore pellet. Finally, the spore suspension was adjusted to the desired concentration (approximately 10^8^ CFU/mL) with sterile distilled water and stored at 4 °C [22].

### 2.3. Preparation and Optimization of Germinants

Germinants that induce spore germination are mainly divided into nutritional germinants and non-nutrient germinants. Nutrient germinating agents include, but are not limited to, L-alanine (L-ala), L-valine (L-val), and AGFK (consists of L-asparagine, D-glucose, D-fructose, and potassium ions) [23]. The safety of non-nutritive germinants (e.g., DPA) remains a challenge when applied to food matrices.

Adapting Xie’s method [17] of germinant screening, the spore suspension was resuspended in sterile water and adjusted to a concentration of approximately 5 × 10^7^ CFU/mL. Germination was initiated by the addition of different concentrations of nutrient germinants (L-ala, L-val, AGFK (a mixture of L-asparagine, D-glucose, D-fructose, and KCl)), and sterile physiological saline with a pH of 4 was used as a blank control. In the experimental group with the non-nutritive germinant (NaCl), sterile water at pH 4 was used as the blank control. Different kinds of germinants (200 μL) with different concentrations, with a final pH of 4, were added to the 96-well plate, followed by 50 μL of spore suspension. The solutions were incubated at 45 °C in a microplate reader for 4 h, and the OD_600_ value was measured every 10 min. The degree of AAT spore germination is expressed as the percentage reduction in the OD_600_ value (with the initial reading taken as 100%), calculated with the following formula:

[(OD_0_ − OD_t_)/OD_0_] × 100% (1)

OD_0_ is the initial absorbance of the treated group, and OD_t_ is the absorbance of the treated group during incubation. Each experiment was conducted in triplicate.

### 2.4. Inactivation of AAT Spores by PEF Combined with Germinants

Spores were germinated for 2, 3 and 4 h respectively after the addition of the optimized germinants to the spore suspension (final concentration of about 10^7^ CFU/mL, with 20 mL in volume), followed by treatment under PEF at low electric field (electric field strength of 0.18 kV/cm, frequency of 10 Hz, pulse width of 10 μm) for different times (90 s, 120 s, 150 s, 180 s, 210 s). Spores without PEF treatment were used as controls. The temperature was recorded throughout the process.

To determine the effect of PEF in combination with germinant on AAT spores, surviving spores were analyzed with a viable plate counting method before and after treatment [24]. Each sample (1.0 mL), which was serially diluted with 9 mL of sterile water, was spread-plated on an AAM agar plate, and the plates were incubated at 45 °C for 24 h. All treatments were performed in triplicate. Equation (2) was used to calculate the reduction in spores in the experiments:

Y = Log_10_ (N_1_/N_0_) (2)

where Y is the logarithm of spore inactivation after treatment under different conditions, N_1_ is the concentration of spore suspension after treatment under different conditions (CFU/mL), and N_0_ is the concentration of untreated spore suspension (CFU/mL).

To more intuitively compare the AAT spore inactivation rate, the number of surviving spores after inducing germination and PEF treatment was linearly fitted by Equation (3), and the decimal reduction time (D-value) was calculated. The D-value is defined as the time required for a microbial population to decrease by one logarithmic cycle, which can be determined by Equation (4). Results are expressed in seconds.

(3) $$\mathrm{lg}\frac{N}{N_{0}}=-\frac{t}{D}$$

(4) $$D=\frac{t}{\log N_{0}-\log N}$$

where *t* is the treatment time, *N*_0_ is the initial AAT spores’ population, and *N* is the final AAT spores’ population.

### 2.5. Mechanism of Inactivation of AAT Spores by PEF Combined with Germinants

Based on preliminary experimental results, PEF treatment for 210 s after 4 h of incubation with a germinant (40 mM L-val) in the spore suspension minimized the number of spore logs [17]. Therefore, these treatment settings were chosen to determine the mechanism of spore germination.

#### 2.5.1. PI Staining Analysis

PI fluorescent dyes exclusively label cells with damaged membranes, fluorescing red, serving as a reliable marker for inner membrane and cortex damage during spore inactivation. Spores before and after PEF treatment were exposed to 15 μM propidium iodide (PI, Thermo Fisher Scientific, Waltham, MA, USA) fluorescent dye for 15 min away from light for staining, and then centrifuged at 6000 rpm at 4 °C for 8 min to remove excess PI dye. The stained samples were observed and photographed under the confocal laser scanning microscopy (CLSM, Olympus BX61 Microscopes, Tokyo, Japan), with the excitation laser of 488 nm and the emission fluorescence of 645 nm, in order to analyze the structural changes in the spores after different PEF treatment times.

#### 2.5.2. Measurement of Nucleic Acid and Protein Release

DNA and protein are the main substances released by the spores that can absorb ultraviolet light, reaching the maximum absorption peaks at 260 nm and 280 nm, respectively. This reflects the changes in the permeability of the inner membrane of the spores. The suspensions of spores before and after PEF treatment were centrifuged at 8000× *g* for 10 min, and the absorbance of the supernatant at 260 nm and 280 nm was measured by UVspectrophotometer, respectively. Sterile water was used as a blank control, and the untreated group was used as a positive control.

#### 2.5.3. Particle Size Determination

The particle size of the spores before and after PEF treatment was determined by the nanoparticle size and zeta potential analyzer, with the aim of analyzing the extent of disruption of the whole spore structure by the pulsed electric field treatment. Referring to the experimental method of Fan et al., with minor modifications [25], the nanoparticle size and zeta potential analyzer was preheated for 30 min in advance, and the container was cleaned with distilled water before the experiment. The container was filled with the suspension of treated or untreated spores (about 1 mL), and the average size of the spores was determined according to the configured parameters; the average size of the spores in the untreated group was used as a control group.

#### 2.5.4. Scanning Electron Microscopy

Changes in the morphology and structure of spores before and after PEF treatment were observed by scanning electron microscopy. Sample pretreatment steps for SEM included cell washing, fixation, dehydration, drying, and gold sputtering [26]. The details are as follows: AAT spores before and after PEF treatment were centrifuged (8000× *g*, 5 min, 4 °C) and washed twice with PBS (the supernatant was discarded). Spores were fixed in PBS buffer (0.01 M phosphate, pH 7.2) with 2.5% glutaraldehyde at 4 °C overnight. Fixed spores were washed three times with PBS buffer, centrifuged, and dehydrated sequentially in 30%, 50%, 70%, 80%, 90%, and 100% ethanol solutions. Subsequently, the dehydrated samples underwent tert-butanol treatment for solvent substitution, followed by natural drying on tinfoil. The thoroughly dried samples were then sputter-coated with gold for approximately 2 min, and the morphological alterations of spores were observed by scanning electron microscopy (SEM).

#### 2.5.5. Na^+^/K^+^-ATPase Activity Assay

Na^+^/K^+^-ATPase is a carrier protein, also known as a sodium-potassium pump, that resides on the plasma membrane and regulates the balance of Na^+^ and K^+^ concentrations. This is important for cellular growth and metabolism. Determination of the Na^+^/K^+^-ATPase activity of the spores after PEF treatment was performed according to the method of Li et al., with minor modifications [27]. The samples were collected and broken by ultrasonication (power 200 W, ultrasonication for 3 s, interval 10 s, and repeated 30 times) before and after PEF treatment. The spores were then centrifuged, and the supernatant was taken for enzyme viability assay analysis using the Na^+^/K^+^-ATPase assay kit.

#### 2.5.6. Intracellular Reactive Oxygen Species Concentration Detection

ROS levels within the AAT spores were quantified by a slight modification of the method described by Ma et al. [28]. 2,7-dichlorodihydrofluorescein (DCFH, a cell-permeable agent that can be hydrolyzed and oxidized by intracellular ROS, generating fluorescence signals) was used as an indicator of intracellular reactive oxygen species. The suspension of AAT spores, which were processed with different pulsed electric field processing times, was centrifuged at 6000× *g* for 8 min at 4 °C, and the AAT spores were collected with the centrifuge tubes after the supernatant was discarded, followed by re-suspension in DCFH solution at a concentration of 10 μmol/L.

The suspension of AAT spores with the indicator was incubated in the dark at 45 °C for 20 min and washed with PBS three times to remove free DCFH. The fluorescence intensity of the supernatant was measured in a microplate reader, and the excitation and emission wavelengths were set at 488 nm and 525 nm, respectively. The untreated samples were used as the control group, and their ROS content was taken as 1 (for normalization). The ROS content of each treatment group was converted by the ratio of fluorescence intensity of the treatment group to that of the control group.

### 2.6. Statistical Analyses

Each treatment process was performed at least in triplicate, and the data were statistically analyzed using GraphPad Prism 8 (GraphPad Software, San Diego, CA, USA). The results are presented as the mean ± standard deviation. Subsequently, an analysis of variance (ANOVA) was conducted, followed by Tukey’s test, utilizing SPSS 22.0 software (IBM, New York, NY, USA). Significance was determined at the *p* < 0.05 level.

## 3. Results and Discussion

### 3.1. Germinant Optimization

**Figure 1:**
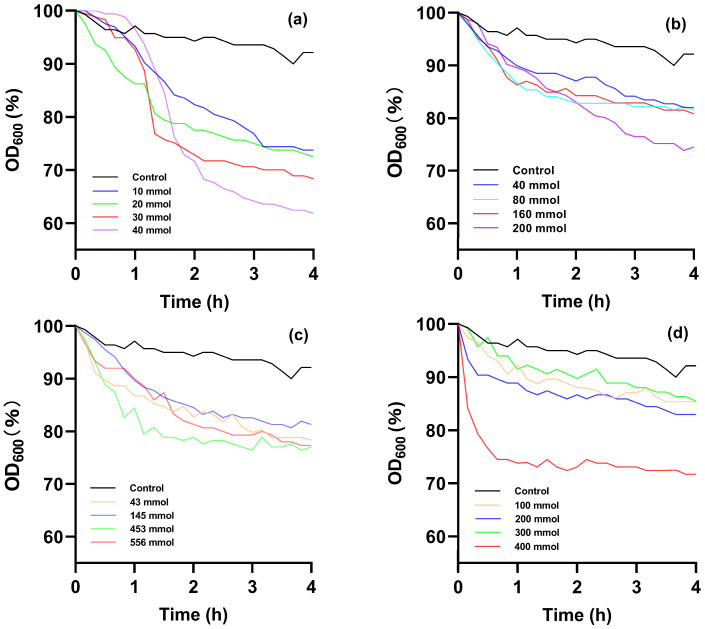
The ratio of OD_600_ values for AAT spores over time during a four-hour incubation period under conditions of varying concentrations of L-valine (**a**), L-alanine (**b**), sodium chloride (**c**), and AGFK (**d**).

Optimizing the type and concentration of germinants is crucial due to the distinct germination properties and requirements of spores from different genera. During germination, the optical density at 600 nm (OD_600_) gradually decreases up to 60% of the initial OD_600_ (i.e., a reduction of about 40%). Therefore, the spore germination can be determined by tracking the changes in OD_600_ [8]. As shown in Figure 1, different types and concentrations of germinants exert varying effects on the change in OD_600_ ratio for AAT spores. In the experimental groups, the OD_600_ of AAT spore suspensions exhibited a significant decline compared to the control group, eventually stabilizing over time. The most effective germinants for inducing AAT spore germination were ranked as follows: L-val > AGFK > L-ala > NaCl > control, suggesting that nutrient germinants are more effective in promoting germination. Nutrient germinants primarily interact with specific receptors located in the inner spore membrane to initiate spore germination. Different germination receptors exhibit specific recognition capabilities for germinants, which consequently results in varying germination performances of spores under the induction of distinct nutritional germinants.

In the group treated with the nutrient germination agent L-ala, the OD_600_ decreased to 74.51% of the control group’s value. Notably, varying concentrations of L-ala did not significantly influence the rate of OD_600_ reduction for AAT spores. Moreover, the germination-inducing effect of L-ala was less pronounced than that observed in the AGFK and L-val experimental groups, which is consistent with previous studies [8,17].

There was no significant difference in the decline rate of OD_600_ for AAT spores when the concentration of the combined nutrient germination agent AGFK was 100, 200, or 300 mM. However, when the concentration was increased to 400 mM, the OD_600_ decreased rapidly and stabilized after 40 min. This suggests that under the influence of 400 mM AGFK, AAT spores entered the germination state more quickly, with a greater germination effect than the L-Ala experimental group. This improvement may be attributed to the ability of AGFK to be recognized by a greater number of germination receptors [29].

The optimal germination-promoting effect was achieved with 40 mmol/L of the nutrient germinant L-Val, which reduced the OD_600_ value of AAT spores to 61.85% after incubation at 45 °C for 4 h. This effect may be attributed to the presence of GerA, a germination receptor in AAT spores that specifically recognizes L-Val. Previous studies have demonstrated that L-Val activates the GerA receptor in Bacillus subtilis spores, thereby inducing germination [30].

Since Na^+^ plays a crucial role in amino acid transport, NaCl has a certain role in inducing germination. However, the NaCl-treated group exhibited the weakest AAT spore germination, with the OD_600_ decreasing by only 77.02%. Based on these findings, L-val at a concentration of 40 mmol/L was selected for further experiments, in combination with PEF treatment, to investigate the inactivation effect and its underlying mechanisms. L-val is a naturally occurring amino acid widely present in various food ingredients such as fruit juices, dairy products, and legumes [31]. Its natural concentration varies by food type and is typically low. However, exogenous addition of amino acids to enhance functional properties is a common practice in food processing, and many countries and regions permit specific amino acids as food additives. Although a 40 mM concentration may exceed natural levels in certain foods, this dosage is achievable through formulation adjustments in food ingredients.

### 3.2. The Inactivation of PEF on AAT Spores

**Figure 2:**
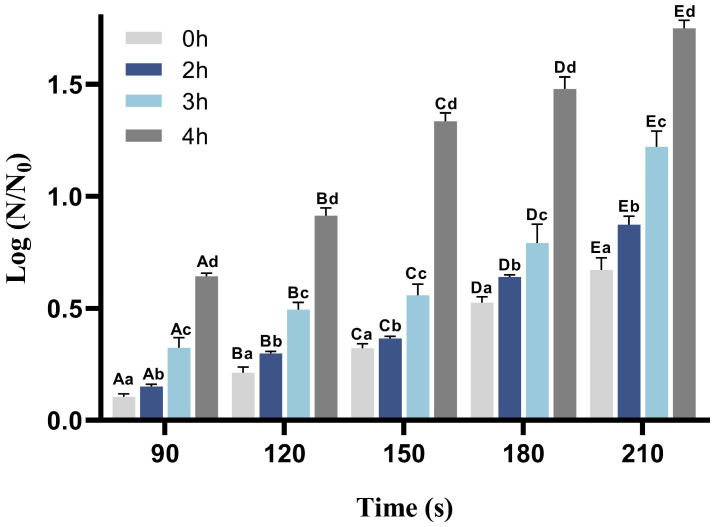
Logarithm of inactivation of AAT spores at different PEF treatment times after inducing germination with 40 mM L-val for different times. Different letters indicate significant differences. Upper case letters indicate significant differences between groups (*p* < 0.05) and lowercase letters indicate significant differences within groups (*p* < 0.05).

**Figure 3:**
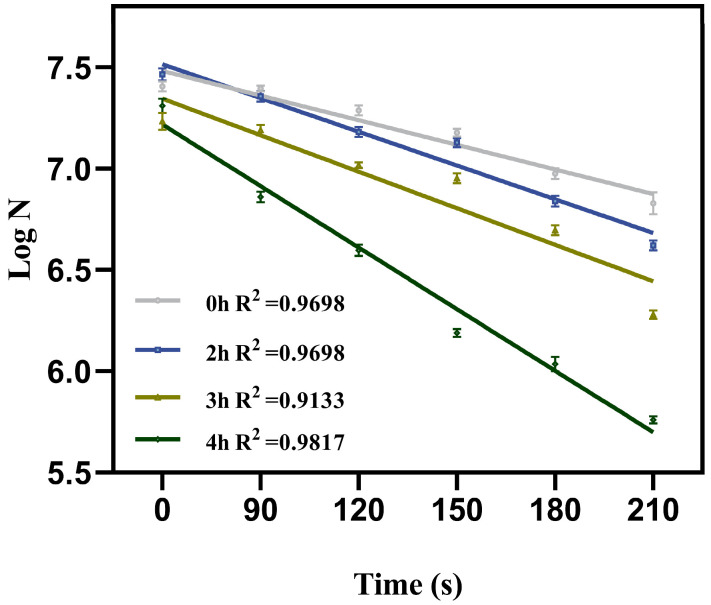
Survivor data of AAT spores treated with PEF after inducing germination with 40 mM L-val for different times and fitted by Equation (3).

The inactivation efficacy of low-intensity pulsed electric field (PEF) treatment combined with germinants on AAT spores is shown in Figure 2. After incubating the spore suspension with the nutrient germinant L-valine (40 mM) for varying durations (2, 3, and 4 h), PEF-induced spore inactivation increased progressively with longer incubation times under consistent treatment parameters. This enhancement is likely due to prolonged exposure to the germinant, which facilitates the transition of AAT spores into the germination phase, resulting in a significant reduction in their resistance. The maximum log reduction (1.733 ± 0.0075) of AAT spores was achieved after 210 s of PEF treatment under optimized conditions (0.18 kV/cm electric field strength, 10 Hz pulse frequency, and 10 μs pulse width). Figure 3 displays the survival data of spores subjected to PEF treatment for varying durations following different germination times induced by 40 mM L-valine. As shown, the data closely fit the linear Equation (3), with all R^2^ values exceeding 0.9. The spore reduction rate (D-value) is positively correlated with the duration of germination induction. Spores without induction required 311.14 s of low-field-strength PEF treatment to achieve a one-logarithm reduction. In contrast, spores induced with 40 mM L-valine for 4 h required only 118.71 s to reach the same level of reduction. As mentioned above, the PEF-germinant synergy notably enhanced spore inactivation under low-intensity electric fields, with a 4-h germinant induction period effectively reducing spore resistance and improving PEF efficiency under sublethal electric field conditions.

**Figure 4:**
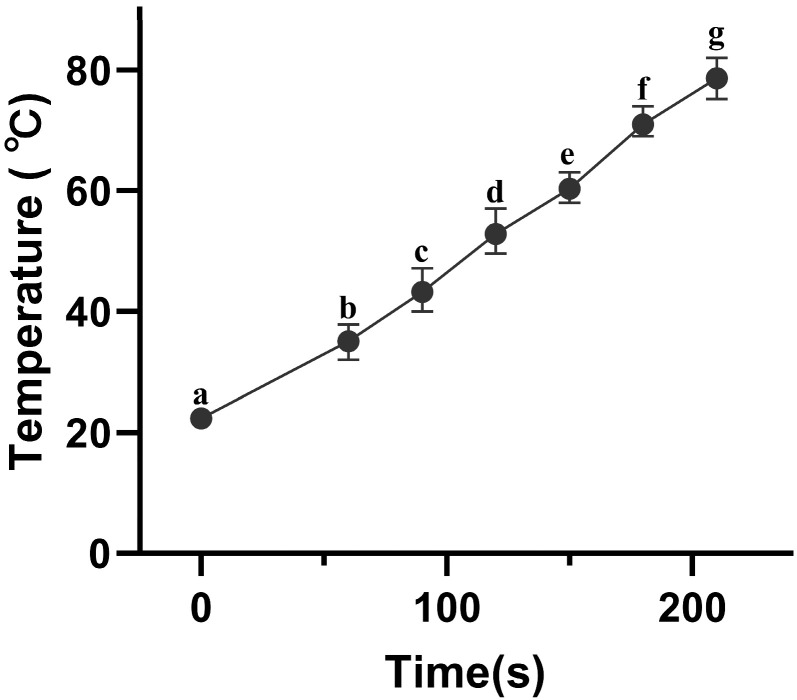
Time-temperature profiles of the AAT spore suspension exposed to PEF. Different letters represent significant differences (*p* < 0.05).

Notably, the inactivation efficiency exhibited a trend of increasing over time. While this was partly attributed to reduced spore resistance caused by germination agents, the thermal effect generated during electric field treatment, specifically the contribution of ohmic heating to spore inactivation, which cannot be overlooked. The ohmic heating (OH) effect produced during PEF treatment progressively intensified [13]. Figure 4 displays the heating curve of the AAT spore suspension treated with 210 s of PEF, showing a steady temperature increase over time. This indicates that temperature elevation indeed accompanies the electric field treatment. These results suggest that the inactivation observed when spores are first induced by a germination inhibitor and then treated with a pulsed electric field may result from the synergistic effects of both the electric field and heat. This inference is not unfounded, as the synergistic effect between electric fields and OH has been demonstrated in numerous prior experiments [32,33,34]. For instance, when investigating the inactivation of *Bacillus subtilis* spores by a medium-intensity electric field (MEF, 300 V/cm), Wang et al. [7] established a strict “heat-only” control (no electric field at the same temperature), clearly demonstrating that MEF produces significant additional inactivation independent of pure thermal effects at sublethal temperatures between 55 and 75 °C. The fundamental mechanism was attributed to the structural disruption of the spore coat and inner membrane by the electric field.

Although this study did not include a fully matched isothermal thermal control, the low-intensity PEF treatment (0.18 kV/cm) induced a 1.73-log inactivation while accompanied by a greater temperature rise, strongly indicating that germination pretreatment significantly enhanced spore sensitivity to subsequent “thermo-electrical synergy.” This significantly reduces the required electric field strength for synergistic inactivation, offering novel insights for developing truly low-energy, low-thermal-load gentle sterilization technologies for fruit juices. Future studies will further quantify the specific contribution ratios of heat and electric fields under this novel strategy by designing precise isothermal thermal control experiments.

### 3.3. Structural Damage of AAT Spores by PEF Synergistic Germinant

**Figure 5:**
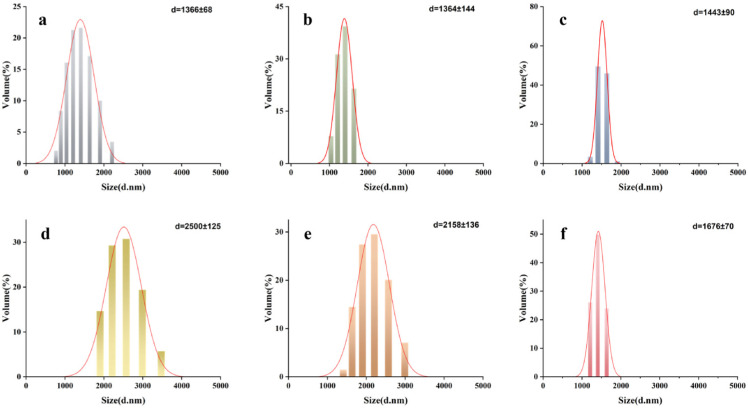
Changes in particle size of AAT spores at different times (0, 90, 120, 150, 180, and 210 s correspond to figures (**a**–**f**), respectively, of treatment under pulsed electric field.

The structural damage patterns observed in this study provide direct evidence for “OH-electric field synergistic” inactivation. As shown in Figure 5, in the particle size analysis of AAT spores subjected to PEF treatment, when the PEF treatment duration ranged from 0 to 150 s, the average particle size of the AAT spores increased, indicating that mild thermal effects and electric field-induced stress responses may have caused spore aggregation. However, as the treatment time further extended (150 to 210 s), the particle size began to decrease and finally reached 1676 ± 70 nm (Figure 5f). This suggests that as treatment time extended, synergistic effects intensified. Specifically, electroporation induced by low-intensity PEF on the sensitized inner membrane of germinated spores, combined with thermal effects, ultimately caused physical disintegration of spore structures, resulting in subsequent particle size diminution.

**Figure 6:**
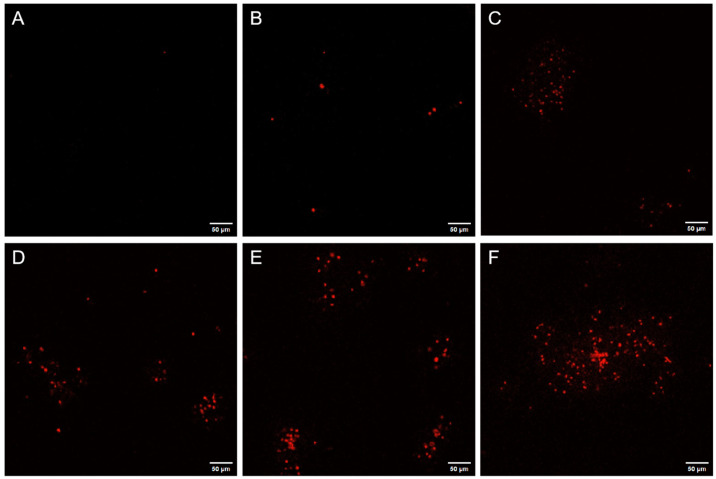
Images of AAT spores produced under pulsed electric field treatment at 0 s (**A**), 90 s (**B**), 120 s (**C**), 150 s (**D**), 180 s (**E**), and 210 s (**F**) observed by confocal laser scanning microscopy.

Spores with damage to the inner or cortical layers, after staining, exhibited red fluorescence under a laser confocal microscope. Figure 6 shows laser confocal images of spore suspensions stained with PI fluorescence following different treatments. AAT spores that were not subjected to PEF treatment did not exhibit red fluorescence under CLSM after 4 h of germination induction, indicating that the spore membrane remained intact. Only a small proportion of AAT spores exhibited red fluorescence after a 90 s low-intensity PEF treatment, suggesting that membrane damage was minimal and the spores retained their biological activity [8]. As the treatment time increased, more spores fluoresced red and gradually aggregated. At 210 s, a significant number of AAT spores exhibited red fluorescence, with damaged spores clustering toward the center, displaying a strong aggregation effect. These observations align with the particle size measurements. Collectively, the results suggested that PEF treatment, following germinant induction, inactivates AAT spores by disrupting the cortex and inner membrane, although the spores increase their resistance through aggregation.

More importantly, CLSM images reveal that not all spores are damaged simultaneously or uniformly. This heterogeneous damage pattern strongly suggests that the dominant mechanism is not uniform thermal denaturation, but rather locally induced membrane perforation triggered by the PEF and dependent on the individual state of the spore. The role of thermal effects in this process may be multifaceted: first, it likely increases the fluidity of inner membrane lipids, lowering the threshold for electroporation; second, it may facilitate the expansion of preformed microoperations and the leakage of contents. Thus, the observed structural damage represents a synergistic amplification between OH and the electric field, rather than the result of a single factor.

**Figure 7:**
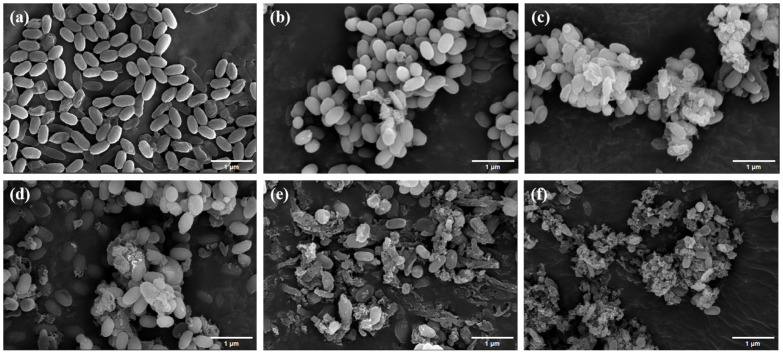
Images of AAT spores under SEM after different treatment times: blank (**a**), 90 s (**b**), 120 s (**c**), 150 s (**d**),180 s (**e**), and 210 s (**f**).

Scanning electron microscopy (SEM) was employed to characterize microstructural alterations in AAT spores subjected to germinant-PEF combined treatment, enabling direct visualization of structural degradation. Figure 7a reveals untreated AAT spores exhibiting characteristic morphology, namely elliptical conformation with intact surface striations and minimal structural compromise, indicating that the spores were morphologically intact and highly resistant.

Most spore morphostructures remained intact after 90 s of PEF treatment (Figure 7b), with a few spores exhibiting deepened folds or sunken perforations. As the treatment duration increased from 120 to 180 s, the spores exhibited intensified morphological damage, transitioning from aggregation and signs of lysis or rupture to extensive structural breakdown, with large areas of rupture and leakage of intracellular material (Figure 7c–e). The presence of numerous spore fragments in Figure 7f indicates that the most significant damage to AAT spores occurred after 210 s of PEF treatment.

Reported studies suggest that irreversible damage to the cortex and inner membrane is likely the primary cause of AAT spore inactivation following PEF treatment, rather than the damage to spore-nutrient germination receptors or cortical lysis enzymes [35]. As early as 2006, Huang et al. observed holes in the spores via scanning electron microscopy (Hitachi S-570 model; 13,000× magnification), which were likely caused by the pulsed electric field [36]. These findings strongly suggest that the electroporation effect induced by PEF disrupts the spores’ multilayered, dense structure, leading to irreversible damage and spore inactivation.

### 3.4. Synergistic Effect of Germinant and PEF Treatment on AAT Spores Membrane Permeability

**Figure 8:**
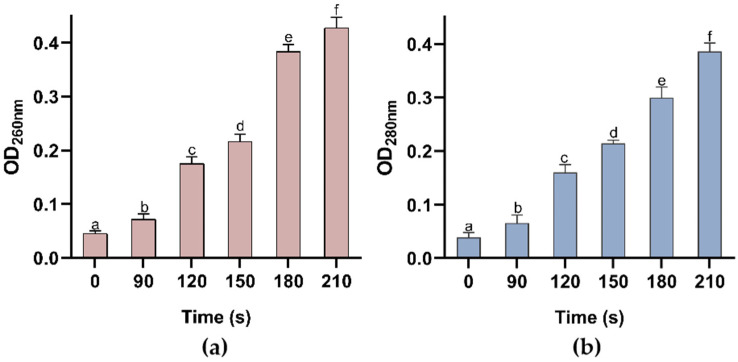
Extracellular nucleic acids (**a**) as well as proteins (**b**) in AAT spores after treatment with pulsed electric fields for different times. Different letters represent significant differences (*p* < 0.05).

Changes in membrane permeability and related metabolic indicators further elucidate the molecular pathway by which the synergistic effect of OH and PEF leads to spore death. Leakage of proteins and nucleic acids is widely recognized as a reliable indicator of alterations in cell membrane permeability. Figure 8 demonstrates the synergistic effect of germinant and PEF treatment on the leakage of extracellular protein and nucleic acid content from *Alicyclobacillus acidoterrestris* (AAT) spores. In the control group (0 s of PEF treatment), the extracellular concentrations of nucleic acids and proteins were relatively low, with OD_260nm_ and OD_280nm_ values of 0.044 ± 0.0047 and 0.036 ± 0.0092, respectively, suggesting that even with the addition of germinants, the spores can still remain morphologically intact and resistant to damage. In addition, Barsotti et al. confirmed that spores can retain their resistance even after exposure to short-duration pulsed electric fields (with an electric field intensity of 15 kV/cm and a pulse duration of 5 s) [37]. As the duration of low-intensity PEF treatment increased to 210 s, the leakage of proteins and nucleic acids from the spores progressively escalated, resulting in a marked increase in the extracellular protein and nucleic acid content. The absorbance values at 260 nm and 280 nm reached a maximum of 0.422 ± 0.0157 and 0.383 ± 0.0196, respectively. These findings implied that, during the brief PEF exposure, the spores retained their structural integrity, resulting in inefficient inactivation. However, with prolonged exposure, the damage became irreversible, leading to increased leakage of nucleic acids and proteins, which indicated that the initial thermal effects and electric field effects may only cause sublethal damage. Once the synergistic effects exceed a critical threshold, irreversible collapse of membrane integrity occurs [35].

**Figure 9:**
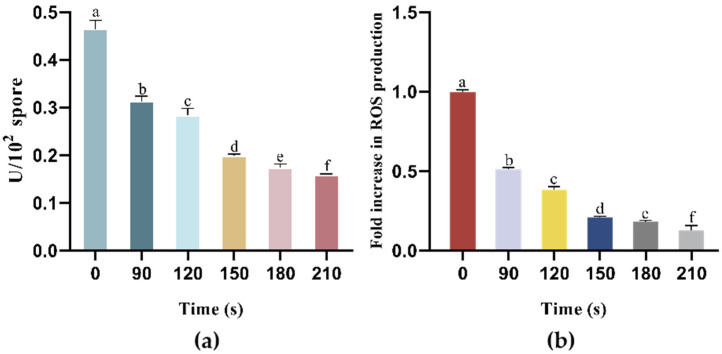
Changes in ATP (**a**) and ROS (**b**) content in the AAT spores after different PEF treatment times. Different letters represent significant differences (*p* < 0.05).

ATPase is a carrier protein located in the plasma membrane that regulates the balance of intracellular sodium and potassium ion concentrations. Moreover, ATPase activity is crucial for maintaining intracellular energy homeostasis, as it reflects the efficiency of cellular energy metabolism. The observed decrease in ATPase activity in the AAT spores after pulsed electric field (PEF) treatment at various times suggests that PEF treatment can inactivate the ATPase in the spore membrane (Figure 9a). In the control group, the ATPase activity of the spores was 0.469 ± 0.0184 U/10^2^ spores. At this stage, the appropriate concentration of the nutrient germinant L-valine induced the spores to enter the initial phase of germination. Consequently, the spores transitioned from a dormant to an active state, initiating energy metabolism and maintaining ATPase activity at a relatively high level. As the PEF treatment time was increased to 120 and 150 s, ATPase activity decreased to 0.278 and 0.199 U/10^2^ spores, respectively, indicating a significant impact of PEF treatment on ATPase activity. The lowest ATPase activity was observed in the 210 s PEF treatment group, where the activity dropped to 0.159 U/10^2^ spores. Previous studies have shown that intracellular enzyme activity in Escherichia coli was reduced when the pulsed electric field strength exceeded 25 kV/cm. Similarly, research on *Aspergillus niger* spores has indicated that electric field strengths inhibit enzyme activity in the spores, with this effect being irreversible [38]. Moreover, this phenomenon may still result from the synergistic interaction between OH and the electric field. While simple thermal heating can indeed inhibit enzyme activity, the dramatic decline observed in this study is more likely due to the direct leakage or denaturation of enzyme proteins following severe disruption of the membrane system caused by this synergistic effect. In conclusion, after 4 h of germination induction, the PEF electric field significantly affected the activity levels of enzymes within the spores. Even at low intensities, the electric field caused irreversible ATPase inactivation, disrupting energy metabolism, which led to cell death and ultimately increased spore inactivation efficiency [39].

Reactive oxygen species (ROS), including superoxide anions (O_2_^−^), hydroxyl radicals (OH^−^), and hydrogen peroxide (H_2_O_2_), are produced during cellular metabolism. Variations in cellular ROS concentrations can be attributed to several factors, including cell number, environmental stressors, and the cell’s activity or stress state. Environmental changes that induce oxidative stress via ROS can cause damage to vital cellular components, such as proteins and nucleic acids, ultimately leading to apoptosis [40,41]. Figure 9b illustrates the intracellular ROS levels in the AAT spores after pulsed electric field (PEF) treatment for different durations. The ROS level in the control group (0 s) was normalized to 1. After 90 s of PEF treatment, the intracellular ROS level was only 50.7% of that in the control group. With prolonged PEF exposure, the ROS levels in the AAT spores decreased to 38.4%, 20.6%, and 18.5% in the PEF-120 s, PEF-150 s, and PEF-180 s treatment groups, respectively. Finally, after 210 s of PEF treatment, only 12.1% of ROS remained within the cells. These results demonstrated a negative correlation between intracellular ROS levels and treatment duration.

This result differs from reports in some studies indicating that stress leads to elevated ROS levels [42,43]. We speculate that this does not imply the absence of oxidative stress, but rather that the damaging effects of PEF on spore structure predominate. As demonstrated by PI staining and SEM results, prolonged PEF treatment caused irreversible damage to the spore inner membrane. Upon loss of membrane integrity, intracellular small molecules, including ROS, will leak out in large quantities. Therefore, the net decrease in intracellular ROS we observed likely resulted from leakage rates through damaged membranes exceeding the rate of new ROS production within cells. This mechanism also aligns perfectly with the time-dependent increase in membrane permeability confirmed by nucleic acid and protein leakage data. Similar results were reported in the study by Cai et al., where the intracellular ROS level in *Alicyclobacillus* spp. DSM 3922 cells treated with a high-voltage PEF decreased to 0.44 times that of the untreated control [44]. Furthermore, the loss of spore structure allows DCPH to be expelled during centrifugation and similar operations, resulting in reduced fluorescence intensity in the treatment group. Thus, the decrease in intracellular ROS levels following PEF treatment can be interpreted as a marker of severe disruption to the spore membrane system and leakage of contents, ultimately leading to metabolic dysfunction and spore death.

## 4. Conclusions

**Figure 10:**
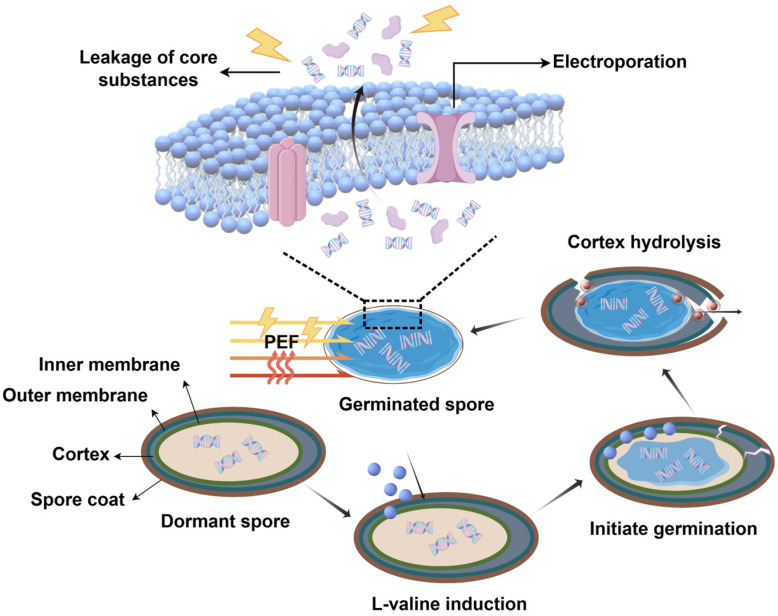
Mechanism of the inactivation of AAT spores by pulsed electric fields.

In this study, the “germinate-to-inactivate” strategy was successfully applied in conjunction with low-intensity PEF treatment. This approach effectively inactivated AAT spores while operating at a substantially reduced electric field strength (0.18 kV/cm), highlighting its potential for lower energy consumption. Systematic optimization identified 40 mM L-valine as the most effective germinant, inducing spore germination and significantly diminishing their inherent resistance. The subsequent low-intensity PEF treatment, while accompanied by OH to a maximum of 78.7 °C, achieved an inactivation of 1.73 log. Crucially, the observed lethality is attributed to a synergistic effect between the low-strength electric field and mild heating, rather than to a purely non-thermal mechanism. This synergistic action was dramatically potentiated by the germination pre-treatment, which sensitized the spores. The underlying mechanism involves PEF-induced electroporation, likely facilitated by the thermal effect on membrane properties, leading to irreversible damage to the inner membrane and cortex, which was confirmed through direct visualization (SEM, CLSM) and was manifested as the leakage of intracellular components (DNA, protein), a collapse in metabolic activity (decreased ATPase), and the efflux of ROS (as shown in Figure 10). While the specific quantitative contribution of thermal versus electric effects warrants further investigation through precise isothermal controls, this study clearly demonstrates that germinant-assisted, low-field-strength PEF offers a novel and enhanced inactivation pathway. It provides a promising avenue for developing gentle processing technologies that aim to balance microbial safety with the preservation of food quality in heat-sensitive products like juices. However, translating this method to real juice applications necessitates considering potential interactions with key matrix components—such as soluble solids (e.g., sugars and pectins), natural acidity, and electrical conductivity—which may alter the inactivation efficacy observed in model systems. For instance, the higher ionic strength of juices could attenuate the applied electric field, while organic acids might exhibit synergistic effects with PEF [45,46]. These factors could lead to significant differences in spore inactivation performance compared to controlled buffer solutions, and thus warrant further investigation. Concurrently, future research should continue to focus on elucidating the molecular interactions between electric fields and key spore structures at this low-intensity regime, and on optimizing germinant formulations to maximize synergy and practical applicability.

## Data Availability

The original contributions presented in this study are included in the article. Further inquiries can be directed to the corresponding authors.

## Abbreviations

The following abbreviations are used in this manuscript:

PEF	Pulsed Electric Field
OH	Ohmic heating
AAT	Alicyclobacillus acidoterrestris
